# Low-dose CT measurements of airway dimensions and emphysema associated with airflow limitation in heavy smokers: a cross sectional study

**DOI:** 10.1186/1465-9921-14-11

**Published:** 2013-01-28

**Authors:** Akkelies E Dijkstra, Dirkje S Postma, Nick ten Hacken, Judith M Vonk, Matthijs Oudkerk, Peter MA van Ooijen, Pieter Zanen, Firdaus A Mohamed Hoesein, Bram van Ginneken, Michael Schmidt, Harry JM Groen

**Affiliations:** 1 University of Groningen, Department of Pulmonary Diseases, University Medical Center Groningen, GRIAC research institute, Groningen, the Netherlands; 2 University of Groningen, Department of Epidemiology, University Medical Center Groningen, Groningen, the Netherlands; 3 University of Groningen, Department of Radiology, University Medical Center Groningen, Groningen, the Netherlands; 4 Division of Heart & Lungs, University Medical Center Utrecht, Utrecht, the Netherlands; 5 Department of Radiology, Radboud University Nijmegen Medical Centre, Nijmegen, the Netherlands; 6 Fraunhofer MEVIS, Institute for Medical Image Computing, Bremen, Germany

**Keywords:** Airway dimensions, Low-dose CT, Respiratory symptoms, Smoking, Airflow limitation, Emphysema

## Abstract

**Background:**

Increased airway wall thickness (AWT) and parenchymal lung destruction both contribute to airflow limitation. Advances in computed tomography (CT) post-processing imaging allow to quantify these features. The aim of this Dutch population study is to assess the relationships between AWT, lung function, emphysema and respiratory symptoms.

**Methods:**

AWT and emphysema were assessed by low-dose CT in 500 male heavy smokers, randomly selected from a lung cancer screening population. AWT was measured in each lung lobe in cross-sectionally reformatted images with an automated imaging program at locations with an internal diameter of 3.5 mm, and validated in smaller cohorts of patients. The 15^th^ percentile method (Perc15) was used to assess the severity of emphysema. Information about respiratory symptoms and smoking behavior was collected by questionnaires and lung function by spirometry.

**Results:**

Median AWT in airways with an internal diameter of 3.5 mm (AWT_3.5_) was 0.57 (0.44 - 0.74) mm. Median AWT in subjects without symptoms was 0.52 (0.41-0.66) and in those with dyspnea and/or wheezing 0.65 (0.52-0.81) mm (p<0.001). In the multivariate analysis only AWT_3.5_ and emphysema independently explained 31.1%and 9.5%of the variance in FEV_1_%predicted, respectively, after adjustment for smoking behavior.

**Conclusions:**

Post processing standardization of airway wall measurements provides a reliable and useful method to assess airway wall thickness. Increased airway wall thickness contributes more to airflow limitation than emphysema in a smoking male population even after adjustment for smoking behavior.

## Introduction

The quantification of airway dimensions by CT has become feasible with the development of multi detector computed tomography (CT) and new software tools for image analysis [1,2]. Assessment of airway dimensions by CT has been studied particularly in relation to asthma, smoking and chronic obstructive pulmonary disease (COPD) [3-8], diseases generally associated with chronic or intermittent airflow limitation. So far, airway wall thickness (AWT) measurements have been performed by selecting well quantifiable airways [9-12] or by standardizing dimensions to airways with a 10 mm internal lumen perimeter (pi10, equivalent to about 3.2 mm internal airway lumen diameter) derived from a small number of airways [5,6,13-15].

More recently, low-dose multi slice CT has become available, a technique with a good quantifying performance that is preferred for monitoring of pulmonary and airway abnormalities as compared to the high radiation exposures with high-resolution computed tomography (high-dose CT). The cumulative radiation dose exposure with low-dose CT remains very low, even when individuals are exposed multiple times [16]. Airway dimensions measured with low-dose CT have only been reported in few studies using the same diversity in analytic approaches as applied with high-dose CT measurements [7,17-19].

Airway dimensions and extent of emphysema are known to be associated with airflow limitation [6,9-14,19,20], although the influence of smoking behavior or signs of airway disease such as cough, dyspnea, wheezing or mucus overproduction on airflow limitation is not clear [5,7,21,22].

The aims of this study are to quantify airway dimensions of the lung in multiple airway sections of each lobe in a novel manner and the extent of emphysema by using low-dose CT. These measurements were associated with the influence of airflow limitation, respiratory symptoms and corrected for smoking behavior.

## Methods

### Population

We randomly selected 500 current and former smokers participating in the Groningen cohort of a male population-based multi-centre lung cancer screening study (NELSON). The Dutch ministry of health and the Medical Ethics Committee of the hospital approved the study protocol. Informed consent was obtained from all participants. Detailed inclusion criteria and characteristics have been described elsewhere [23]. In short, subjects with a smoking history of at least 20 pack-years were included. Information on the presence or absence of respiratory symptoms and smoking (pack-years and former or current smoking) was obtained by questionnaires. The question used to record respiratory symptoms was “do you have experienced the following symptoms cough, sputum expectoration, wheezing or dyspnea for at least 3 months during the past year, even when you did not have a cold?”

### Lung function

All participants performed standardized spirometry according to the European Respiratory Society guidelines [24], including forced expiratory volume in 1 sec (FEV_1_) and forced vital capacity (FVC) at the start of the study. In this population- based study we did not administer a bronchodilator.

### CT scanning

Low-dose CT images of the chest were acquired at full inspiration after appropriate instruction on one CT scanner (Sensation 16, Siemens Medical Solutions, Forchheim, Germany) [23] according to the following protocol: spiral acquisition at 120 kV, 20mAs, rotation time 0.5 s, pitch 1.5 and collimation 16×0.6 mm, field of view 300 to 350 mm, slice thickness 1 mm and slice increment 0.7 mm. The effective radiation dose was less than 0.8 mSv. Contrast medium was not used. The images were reconstructed to a pixel matrix of 512×512 using B30f kernel. Thus, the spatial resolution was 0.59 to 0.68 mm in *xy* plane, and 0.7 mm in *z* plane. The CT system was calibrated routinely.

### Quantification of AWT

AWT was measured in cross-sectionally reformatted images with an automated research software prototype MEVIS Airway Examiner v1.0 (release 2009, Fraunhofer MEVIS, Bremen, Germany) based on an algorithm by Weinheimer at locations with a fixed internal diameter of 3.5 mm in each lung lobe [25]. This software automatically extracts airway centerlines, re-samples images perpendicular to the airway direction at equally spaced positions along the centerline and detects inner and outer airway wall borders in these images. The outer wall border is detectable when no adjacent tissue with similar CT density is present and is taken into account when the wall is detected in at least 25% of the perimeter at a location. AWT and the fraction of perimeter where the outer wall border was identified (Assessed Perimeter Fraction, APF) are calculated for each location. Wall thickness quantification accounts for partial volume effects by integrating Hounsfield units across the wall. Accuracy and reproducibility of this algorithm was tested previously under clinical conditions using a similar protocol as used in our study [2]. Average wall thickness and cumulative APF of all detectable airway locations with a fixed lumen diameter is reported per lobe and for the whole lung. The borders of the lung lobes were automatically calculated by the software in a standard way. All low-dose CT scans were visually evaluated for appropriate segmentation.

### Quantification of emphysema and lung volume

Quantification of emphysema was based on density differences and measured with a software tool called Image Xplorer (Image Sciences Institute, Utrecht, the Netherlands) [16,26]. This software produces automatically the lung volume. The extent of emphysema was automatically performed at the 15^th^ percentile (Perc15) of the Hounsfield density distribution. Perc15 is the threshold density value where 15% of all voxels has a lower density [27]. A lower Perc15, i.e. closer to −1000 HU, means that more emphysema is present. All scans were reconstructed with a soft reconstruction filter (Philips B, Siemens B30f). Airways were automatically excluded to assess density of lung parenchyma exclusively and HU densities of the entire scan were recalibrated using automatically measured average densities in the trachea and shifting the HU values of the entire scan so that air density in the trachea became −1000 HU. Additionally, the percentage of low attenuation area, defined as the proportion of low-density voxels below −950 HU (%LAA-950HU) was used. %LAA-950HU was log-transformed because of skewed distribution.

### Explorative studies

Prior to the research described above we have

1) established the optimal internal airway diameter, i.e. the internal airway diameter that allows the highest number of cumulatively Assessed Perimeter Fractions (APF) for the whole lung. Therefore we measured APF on 20 selected NELSON CT’s in airways with a lumen diameter of 2.5, 3.0, 3.5, 4.0, 4.5 and 5.0 mm (± 0.25 mm) divided into 3 groups: no emphysema and normal lung function (n = 8, p^15^ > −920 and FEV_1_/FVC > 85 %), moderate emphysema and normal lung function (n = 4, −940 < p^15^ < −960 and FEV_1_/FVC > 70 %) and no emphysema and severe airflow limitation (n = 8, p^15^ > −920 and FEV_1_/FVC < 50 %).

2) compared the mean AWT_3.5_, using the same method as described above, at the established internal lumen size with high- and low-dose CT in 8 NELSON subjects from whom high- and low-dose CT were available. These CT data were obtained in spiral mode with 16×0.75 mm collimation and in full inspiration with the same scanner (Sensation-16 Siemens Medical Solutions, Forchheim, Germany). Axial images were reconstructed with 1.0 mm thickness at 0.7 mm increments. All scans were reconstructed with a soft reconstruction filter (Siemens B30f) at a 512×512 matrix.

3) determined the generation where airways with the established optimal internal lumen size are present. AWT measurements at 3.5 mm internal lumen size were performed in 57 randomly selected low-dose CTs of NELSON subjects. A multi-planar reconstruction (MPR) was made in each case of the apical upper lobe bronchus (B1) and the posterior lower lobe bronchus (B10). Subsequently the location was projected on the segmentation image. Three-dimensional image moving created the opportunity to observe airways from various directions and to check bifurcations and count airway generations according to the method of Boyden [28].

### Statistical analysis

Data are reported as mean ± standard deviation (SD) or median (25^th^ - 75^th^ percentile) values as appropriate. The mean AWT at 3.5 mm internal lumen size (AWT_3.5_) of all five lobes per case was calculated taking into account the APF per lobe by the following formulae: ((AWT left upper lobe × APF left upper lobe) + (AWT left lower lobe × APF left lower lobe) + (AWT right upper lobe × APF right upper lobe) + (AWT right middle lobe × APF right middle lobe) + (AWT left upper lobe × APF left upper lobe)) / sum of APF of all lung lobes. AWT_3.5_ for the whole population was skewed distributed, therefore we report median AWT and range, and log-transformed AWT was used in the analyses.

Differences between various categories were explored using chi-square tests (dichotomous data), 2-tailed unpaired Student’s t-tests for normally distributed continuous data and Mann-Whitney U-tests for not normally distributed continuous data. The difference in airway wall thickness between lung lobes was assessed with a Wilcoxon signed rank test. Univariate linear regression analyses was used to study the relationships between clinical variables and AWT, and those variables with FEV_1_%predicted. Next, multivariate linear regression analyses were performed on those clinical variables showing significance in the univariate regression analyses. Outcomes of these analyses have been described with beta’s and p-values. Bland-Altman plot was used to analyze the agreement between AWT by high- and low-dose CT [29]. All statistical analyses were performed using SPSS 18.0 for Windows; P-values below 0.05 were considered statistically significant.

## Results

### Population characteristics

After visual evaluation 8 out of the 500 randomly selected subjects were excluded because of (partial) missing of airway segmentation on CT. The mean age of the cohort was 59.4 (± 5.2) years, approximately 59% were current smokers and median pack-years was 34.0 (28.0 - 45.6). More than 51% of the population reported at least one respiratory symptom (Table 1).

**Table 1:** Clinical and demographic characteristics of heavy smokers from the general population cohort


N	492
Age, years	59.4 ± 5.2
Pack-years smoking	34.0 (28.0 - 45.6)
Current smoking,%	59.1
FEV_1_, liter	3.45 ± 0.76
FEV_1_,%predicted	98.2 ± 19.7
FEV_1_/FVC,%	70.0 ± 10.7
Emphysema; Perc15, HU	−920 (−930 to −907)
Emphysema;%LAA −950 HU	2.5 (1.3 - 4.3)
Emphysema; >5%LAA −950 HU,%	19.7
Lung volume on CT, liter	6.5 ± 1.4
Chronic Mucus Hypersecretion,%	29.7
Cough,%	32.7
Dyspnea,%	28.4
Wheezing,%	25.3
No respiratory symptoms,%	48.6

### Airway wall thickness

#### Exploratory analyses

**Figure 1:**
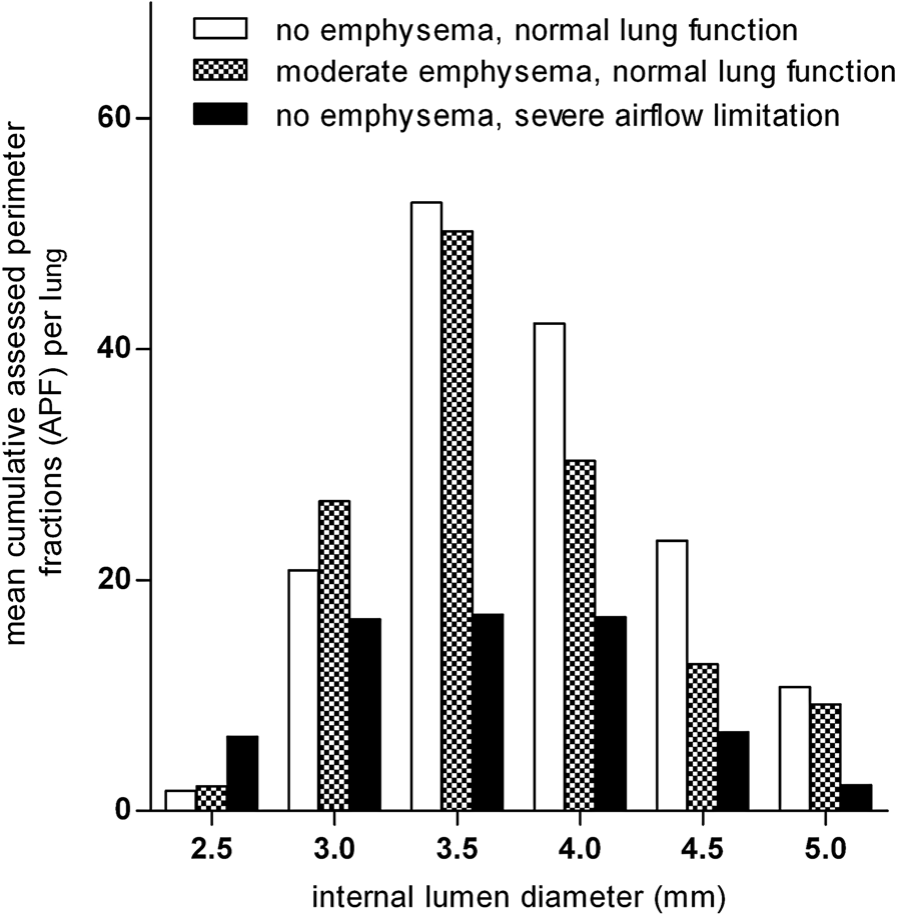
**Determination of the optimal airway size.** Mean cumulative assessed perimeter fractions in the total lung, for different groups of patients at different internal airway lumen diameters. APF was measured on low-dose CT of 20 selected NELSON subjects divided into 3 groups: subjects without emphysema and with normal lung function (n = 8, perc15 > −910 and FEV_1_/FVC > 85 %), with moderate emphysema and normal lung function (n = 4, -940 < perc15 < −960 and FEV_1_/FVC > 70 %) and without emphysema and having severe airflow limitation (n = 8, perc15 > −920 and FEV_1_/FVC < 50 %), in airways with a lumen diameter of 2.5, 3.0, 3.5, 4.0, 4.5 and 5.0 mm (± 0.25 mm).

**Figure 2:**
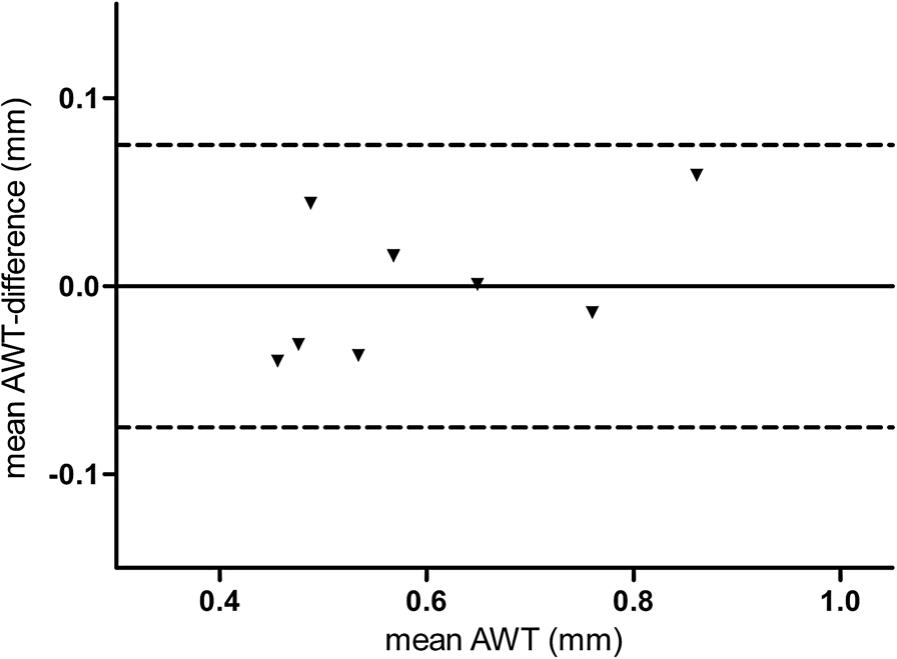
**Comparison of airway wall thickness on high- and low-dose CT.** AWT_3.5_ was measured on high and low-dose CT of 8 NELSON subjects. Bland & Altman plot shows agreement between mean AWT_3.5_ determined by high- and low-dose CT. Dashed lines depict the 95% confidence interval.

**Figure 3:**
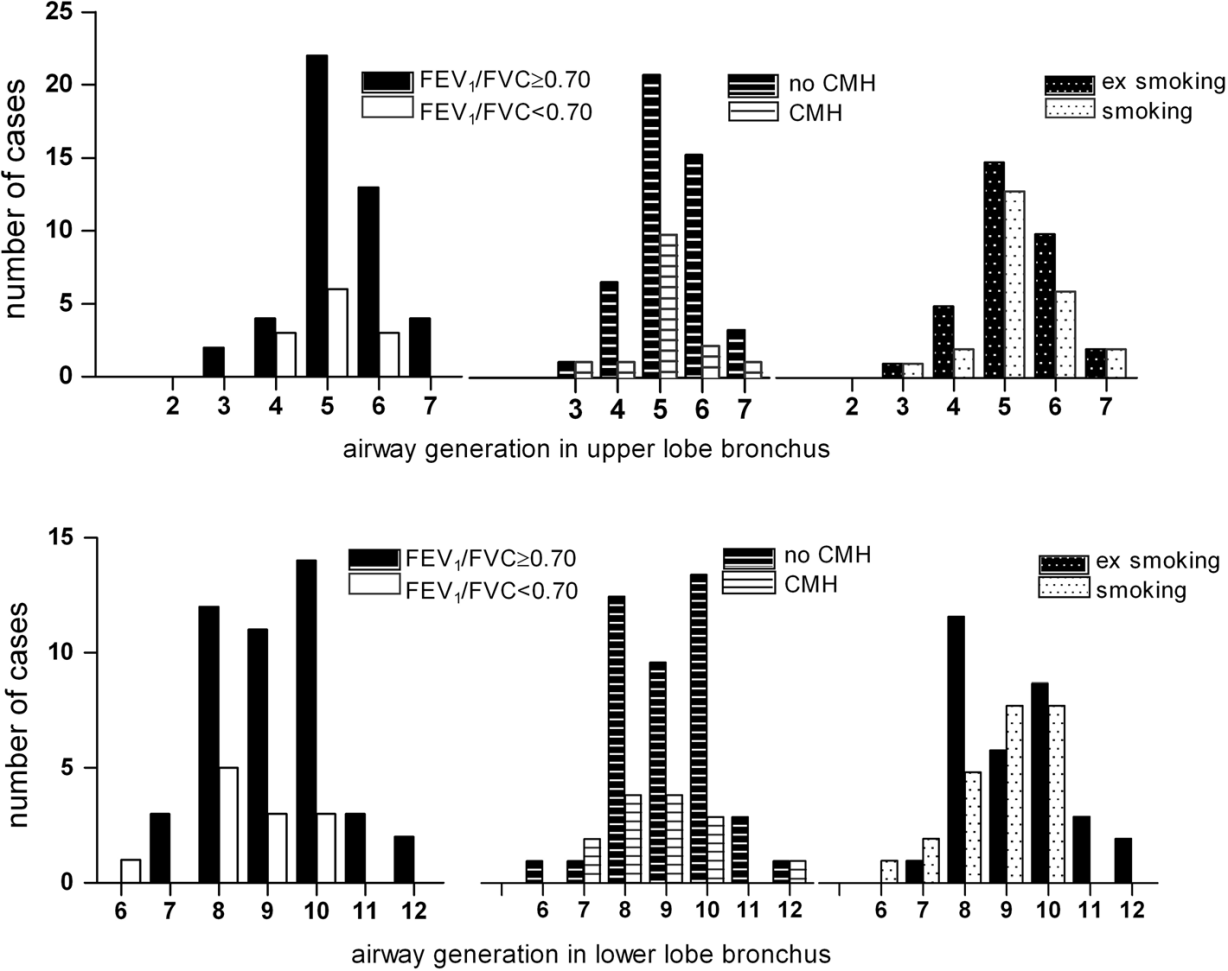
**Distribution of 3.5 mm sized airways over airway generations.** The distribution of 3.5 mm internal lumen sized airways over the 2^nd^ till 7^th^ airway generations in the apical upper lobe bronchus (B1) and over the 6^th^ till 12^th^ generation in the lower lobe bronchus (B10) in the right lung, for subjects with and without airway obstruction, for subjects with and without Chronic Mucus Hypersecretion (CMH) and for current and former smokers. The number of cases (e.g. no CMH and CMH) in each group was limited but the distribution was similar.

The highest numbers of cumulatively assessed perimeter fractions (APF) of airways were reached at an internal lumen perimeter of 3.5 ± 0.25 mm (Figure 1); therefore this diameter was selected for further analysis. Median AWT_3.5_ on low-dose CT was comparable with median AWT_3.5_ on high-dose CT, respectively 0.57 (0.48 - 0.74) mm and 0.55 (0.47 - 0.73) mm (p = 0.89, n = 8). This demonstrates that MEVIS software analysis of data from low-dose CT gives similar results as from high-dose CT (Figure 2). Airways with an internal diameter of 3.5 mm appeared mainly in the 5^th^ generation (range, 3^rd^ - 7^th^ generation) in the upper lobe bronchus, and mainly in the 8^th^ - 10^th^ generation (range, 6^th^ - 12^th^ generation) in the lower lobe bronchus. This distribution was observed irrespective of smoking, presence of airflow limitation (defined as FEV_1_/FVC <0.70) or chronic mucus hypersecretion (CMH) (Figure 3).

### Airway wall thickness in the population

In the whole population the median (25^th^ - 75^th^ percentile) AWT_3.5_ was 0.57 (0.44 - 0.74) mm. The APF in the whole lung varied from 142 to 295 (median 215). The results per lung lobe are presented in the online Additional file 1: Table S1).

### Airway wall thickness and clinical characteristics

Significantly higher AWT_3.5_ values were observed in subjects with dyspnea and/or wheezing (n = 181, median AWT_3.5_ 0.66 mm) or with cough and/or CMH (n = 201, median AWT_3.5_ 0.63 mm) compared to subjects without dyspnea and/or wheezing (n = 309, median AWT_3.5_ 0.53 mm, p<0.001) or without cough and/or CMH (n= 291, median AWT 0.53 mm, p-values < 0.001). Current smokers and former smokers had comparable median AWT_3.5_ values, i.e. 0.58 mm and 0.56 mm (p = 0.113) respectively (Table 2).

**Table 2:** Airway wall thickness in subjects with and without respiratory symptoms and in current and former smokers

	**n**	**AWT**_**3.5**_**(mm)**		**n**	**AWT**_**3.5**_**(mm)**	**p-value**
CMH	146	0.62 (0.49-0.80)	no CMH	346	0.56 (0.44-0.71)	0.002*
Cough	161	0.64 (0.50-0.84)	no cough	331	0.54 (0.43-0.70)	<0.001*
Dyspnea	140	0.65 (0.51-0.80)	no dyspnea	352	0.54 (0.43-0.69)	<0.001*
Wheezing	125	0.66 (0.52-0.85)	no wheezing	367	0.53 (0.43-0.70)	<0.001*
CMH and/or cough	201	0.63 (0.49-0.79)	no CMH or cough	291	0.53 (0.43-0.70)	<0.001*
Dyspnea and/or wheezing	181	0.66 (0.52-0.83)	no dyspnea or wheezing	311	0.53 (0.42-0.67)	<0.001*
Cough, CMH, dyspnea and wheezing	49	0.69 (0.51-0.89)	no cough, CMH, dyspnea or wheezing	239	0.52 (0.41-0.66)	<0.001*
Current smoking	291	0.58 (0.45-0.75)	Former smoking	201	0.56 (0.43-0.71)	0.113

Data presented as median (interquartile range) values.

*Definition of abbreviations:* AWT_3.5_ = airway wall thickness at 3.5 mm internal lumen size; CMH = chronic mucus hypersecretion.

Univariate linear regression analysis showed inverse associations between log-AWT_3.5_ and FEV_1_ (b = −0.233, p < 0.001), FEV_1_/FVC (b = −0.015, p < 0.001), FEV_1_%predicted (b = −0.010, p < 0.001) and lung volume (b = −0.055, p<0.001) and positive associations between log-AWT_3.5_, Perc15 and number of pack-years smoking, respectively (b = 0.003, p < 0.001) and (b = 0.003, p = 0.003) (Table 3).

**Table 3:** **Univariate associations between (A) log-transformed AWT**_**3.5 **_**and (B) FEV**_**1**_**% predicted, and clinical characteristics**

**Dependent variable**	**A. Log-AWT**_**3.5**_		**B. FEV**_**1**_**,% predicted**	
	**Beta**	**p-value**	**Beta**	**p-value**
Log-AWT_3.5_			−31.21	<0.001
FEV_1,_% predicted	−0.010	<0.001		
FEV_1_, liter	−0.233	<0.001	23.56	<0.001
FEV_1_/FVC,%	−0.015	<0.001	1.441	<0.001
FVC,% predicted	−0.009	<0.001	0.971	<0.001
Emphysema; Perc15	0.003	<0.001	0.204	<0.001
Emphysema; Log-%LAA −950 HU	−0.041	0.023	−5.872	<0.001
Lung volume on CT, Liter	−0.055	<0.001	−0.257	0.691
Pack-years	0.003	0.003	−0.153	0.004
Smoking (former/current)	0.059	0.065	−3.257	0.071
Age, years	−0.002	0.458	−0.289	0.092
Height, cm	−0.002	0.444	−0.121	0.425
Cough	0.156	<0.001	−9.234	<0.001
CMH	0.114	0.001	−8.171	<0.001
Dyspnea	0.158	<0.001	−11.99	<0.001
Wheezing	0.202	<0.001	−13.26	<0.001

*Definition of abbreviations:* Log-AWT_3.5_ = log transformed airway wall thickness at 3.5 mm diameter; CMH = chronic mucus hypersecretion; Log-%LAA −950 HU = log transformed percentage of low attenuation areas < −950 HU.

Multivariate analysis on all clinical variables significantly associated with AWT in univariate analyses revealed that log-AWT_3.5_ was independently associated with lower FEV_1_%predicted (b = −0.010, p < 0.001), higher Perc15 (b = 0.005, p < 0.001), and lung volume (b = −0.037, p = 0.005) respectively (Table 4).

**Table 4:** **Multivariate linear regression: dependent variable is (A) log transformed AWT**_**3.5 **_**and (B) FEV**_**1**_**% predicted**

**Dependent variable**	**A. Log-AWT**_**3.5**_		**B. FEV**_**1**_**,% predicted**	
	**Beta**	**p-value**	**Beta**	**p-value**
FEV_1_,% predicted	−0.010	<0.001		
Log-AWT_3.5_			−31.28	<0.001
Emphysema; Perc15	0.005	<0.001	0.342	<0.001
Lung volume	−0.037	0.005	0.209	0.773
Pack-years	0.002	0.031	−0.029	0.499
Smoking (former/current)	−0.011	0.700	−2.756	0.086
Cough	0.030	0.404	−0.408	0.834
CMH	−0.026	0.447	−2.098	0.262
Dyspnea	−0.007	0.828	−2.945	0.116
Wheezing	0.058	0.124	−4.004	0.051

*Definition of abbreviations:* Log-AWT_3.5_ = log transformed airway wall thickness at 3.5 mm diameter; CMH = chronic mucus hypersecretion.

### Contribution of airway wall thickness and emphysema to airflow limitation

To study the contribution of AWT_3.5_, emphysema (Perc15), and clinical variables to airflow limitation, FEV_1_%predicted was taken as dependent variable. A significant negative association was found between FEV_1_%predicted and log-AWT_3.5_ (b = −31.21, p < 0.001), pack-years (b = −0.153, p = 0.004), cough, CMH, dyspnea and wheezing (b = −9.234, −8.17, −11.99, −13.26 respectively, all p-values < 0.001), and a positive association between FEV%predicted and Perc15 (b = 0.204, p < 0.001) (Table 3).

Multivariate analysis on all clinical variables that were associated with FEV_1_%predicted in univariate analyses showed that higher FEV_1_%predicted was significantly associated with lower AWT_3.5_ values (b = −31.3, p < 0.001) and with higher Perc15 (b = 0.342, p < 0.001) (Table 4). AWT_3.5_ and Perc15 explained 31.1% and 9.5% of the variance of FEV_1_%predicted, respectively. The results of the multivariate regression analysis with emphysema expressed as %LAA−950 HU as independent variable, are presented in the online Additional file 1: Table S2.

## Discussion

Low-dose CT is an appealing approach to quantify simultaneously pulmonary and airway abnormalities. Our study shows that the use of low-dose CT combined with modern post processing software tools provides reliable information on airway wall thickness and the extent of emphysema in a heavy smoking male population. Although CT does not provide dynamic measurements, airway wall thickening and emphysema explained respectively 31.1% and 9.5% of the variance in FEV_1_%predicted, the most commonly used variable of airflow limitation. Changes in AWT of more than 0.1 mm reflecting lumen surface changes over 8% measured at one air lumen level were observed between cases with and without respiratory symptoms.

Our study confirmed that increased AWT is associated with lower FEV_1_%predicted. This lower FEV_1_%predicted depends on the airway size in which the measurements of AWT are being performed [12,17] and on the characteristics of the study population [7,20]. Our population consisted of rather healthy elderly males from a randomly recruited Dutch heavy smoking population and still we were able to find significant associations between thicker airway walls and more severe airflow limitation. In contrast with the study of Nakano we found a significant negative association between AWT and FEV_1_/FVC illustrating the sensitivity of our method [9].

The significant but weak negative association between airway wall thickness and emphysema has also been reported in other studies [7,13,30] but was not found in the study by Nakano [9]. Loss of elastic recoil may contribute to collapse of the airways resulting in a more proximal localization of airways with 3.5 mm internal lumen diameter. As these more proximal airways have thicker airway walls this phenomenon contributes to the weak negative association. Another possible explanation for this negative association may be that there are subjects with predominantly airway wall thickening and others with predominantly emphysematous changes. Particularly subjects with relatively more airway wall thickening are responsible for the negative association and subjects with predominantly emphysematous changes do hardly contribute. Apparently, in our population of subjects with normal lung function and with mild airflow limitation, the bronchitic phenotype is already present in the very early stages of smoking-induced lung disease. Discrepancies between the study of Nakano [9] and our study may be due to the composition and size of the study populations, respectively predominantly emphysema versus predominantly healthy smokers with respiratory symptoms.

Importantly, we observed that the contribution of AWT_3.5_ to airflow limitation was larger than the emphysema component. Moreover, AWT_3.5_ and emphysema together only explained about 40% of the variance in FEV_1_%predicted in this smoking male population. This unexpected low contribution of AWT_3.5_ and emphysema to FEV_1_%predicted may be that the CT images were obtained at full inspiration, while FEV_1_ reflects expiratory airflow limitation. One explanation for this observation is that airflow limitation is not only due to reduced airway diameter at one level but should be evaluated as an integral of all airways at all lumen diameters. This is difficult to achieve and therefore we took the smallest measurable lumen diameter that provides the largest contribution to airflow limitation. A more obvious physiological explanation may be the presence of the heterogeneity in airway ventilation interrupting the symmetry in parallel airways leading to large clusters of poorly ventilated lung units [31].

In the univariate analysis, increased AWT_3.5_ was associated with respiratory symptoms. However, AWT_3.5_ was not associated with the presence of any respiratory symptom in the multivariate analysis after adjustment for FEV_1_%predicted, emphysema and smoking behavior. This finding corresponds with other studies [7] and is consistent with the idea that inflammation and airway remodeling, associated with chronic bronchitis, is located in the more central airways [32]. The study of Martinez et al. showed a positive association between airway dimensions and questionnaires, the BODE index [33] and the St. George’s respiratory questionnaire [34] including questions about BMI, respiratory symptoms, exercise capacity and lung function. Also Camiciottoli et al. found a positive association between BODE and airway wall thickness [35]. Our study also showed that including respiratory symptoms in the multivariate model with AWT_3.5_ and emphysema has no impact on airflow limitation.

Lung volume depends on height, weight and sex and as a consequence each person has different airway dimensions. Therefore, airway dimensions should be corrected for lung volume. Actually, volume-corrected AWT is the best parameter to use. In this study lung volume does not change the multivariate model because FEV1%predicted is already corrected for lung volume by correcting for patient height.

It has been shown that the automated imaging program (MEVIS Airway Examiner) based on a method by Weinheimer et al. performed much better than the often used “full-width-at-half-maximum” method in a silicon tube phantom, regarding the blurring effect of CT [25,36]. Usually it is better to use sharper kernels for airway quantification. However, it was shown in the study by Schmidt et al. that the MEVIS airway examiner provides reproducible quantitative results across different reconstruction kernels (B30f and B50f) and repeated acquisitions [2]. Moreover, the “full-width-at-half-maximum” technique systematically overestimates AWT, particularly in small airways [36]. Because low-dose CT and the automated imaging program (MEVIS Airway Examiner) had not been used previously in clinical studies, we firstly optimized our post processing measurements in smaller cohorts of patients before applying it in the population study. We demonstrated that the highest number of AWT measurements could be performed on airways with an internal diameter of 3.5 mm, irrespective of the presence of airflow limitation or emphysema. In addition we demonstrated that differences in AWT_3.5_ are not explained by differences in airway generations. Finally, we demonstrated that low-dose CT imaging provided similar AWT results as high-dose CT imaging.

In a non-biased way we were able to evaluate 230 cumulatively assessed perimeter fractions (APF) per CT, ranging from 27-641 APF. In contrast to the commonly used pi10 method, in which a secondary derived variable from few, mostly 6 selected airways is used to estimate the airway wall thickness [5,6,13-15], we obtained many direct airway wall measurements. To our opinion direct measurements assessed over all lobes provide a better overall reflection of AWT than a limited number of secondary AWT measurements.

A limitation of this study is, inherent to general population-based studies, that only male smokers with mild emphysema and/or airflow limitation were included. Strengths of our study is the non-biased way of analyzing a high number of airway sections in all lobes that makes our approach more suitable for combined airway wall thickness and emphysema measurements on one low-dose CT scan. Such approach allows monitoring of intervention effects on both parameters. This is important when new treatment modalities will become available for clinical testing.

In the future further developments may involve measurements of thickness of airway walls along the full length of the bronchial tree at in- and expiration scans. Possibly more airflow variability will be explained.

In conclusion, post processing standardization of large numbers of airway wall measurements in all lung lobes is feasible, reliable and an useful method to assess airway wall thickness. We have demonstrated that increased airway wall thickness contributes more to airflow limitation than emphysema in a smoking male population even after adjustment for smoking behavior.

## Abbreviations

APF: Cumulative assessed perimeter fractions; AWT: Airway wall thickness; AWT_3.5_: Airway wall thickness at 3.5 mm internal lumen diameter; BMI: Body mass index; CMH: Chronic mucus hypersecretion; CT: Computed tomography; FEV_1_: Forced expiratory volume in 1 sec; FEV_1_/FVC: Ratio of forced expiratory volume in 1 sec and forced vital capacity; FEV_1_%predicted: (Ratio of present FEV_1_ and expected FEV_1_) x 100 %; FVC: Forced vital capacity; GOLD: the Global Initiative for Chronic Obstructive Lung Disease; HRCT: High resolution computed tomography; HU: Hounsfield unit; %LAA: Percentage of low attenuation areas; NELSON: the Dutch-Belgian Randomized Lung Cancer Screening Trial; Perc15: the threshold density value where 15% of all voxels has a lower density; PFT: Pulmonary function test; SD: Standard deviation.

## Competing interests

Dr Postma received funds for research from AstraZeneca, Chiesi, GSK. DSP has been consultant to AstraZeneca, Boehringer Ingelheim, Chiesi, GSK, Nycomed and TEVA. Travel to meetings has been funded by AstraZeneca, Chiesi, GSK and Nycomed. Dr Zanen received an EU FP7 grant. MSc Schmidt received a grant for development of algorithms for the airway examiner by MEVIS Medical Solutions. MSc Dijkstra, Dr ten Hacken, Dr Vonk, Dr Oudkerk, Dr van Ooijen, Dr Mohamed Hoesein, Dr van Ginneken and Dr Groen have reported that no potential conflicts of interest exist with any companies/organizations relevant to this article.

## Authors' contributions

AD designed the study concept, collected data, performed statistical analysis, interpreted data and drafted, wrote and finalized the manuscript; HG and DP designed the study concept, collected data, interpreted data and drafted and finalized the manuscript; NH and FMH interpreted data and drafted and finalized the manuscript; JV performed statistical analysis, interpreted data and drafted and finalized the manuscript; MS designed and supervised the use of software for data collection, interpreted data and drafted and finalized the manuscript. MO, PO, PZ and BG drafted and finalized the manuscript. All authors read and approved the final manuscript.

## Supplementary Material

Additional file 1 ** Table S1.** Median AWT_3.5_ and cumulatively assessed perimeter fractions (APF) per lung lobe. **Table S2.** Multivariate linear regression: dependent variable is (A) log transformed AWT_3.5_ and (B) FEV_1_% predicted. LAA%–950 HU was used to quantify the extent of emphysema in this analysis. Click here for file
