# Supplementary material for: Low-dose CT measurements of airway dimensions and emphysema associated with airflow limitation in heavy smokers: a cross sectional study
Source: Respir Res. 2013 Jan 28;14(1):11. doi: 10.1186/1465-9921-14-11 (PMC3570364; doi:10.1186/1465-9921-14-11)
Supplement: Additional file 1 — Table S1. Median AWT3.5 and cumulatively assessed perimeter fractions (APF) per lung lobe. Table S2. Multivariate linear regression: dependent variable is (A) log transformed AWT3.5 and (B) FEV1% predicted. LAA%–950 HU was used to quantify the extent of emphysema in this analysis. [file 1465-9921-14-11-S1.docx]

Online supplement accompanying the study:

Low-dose CT measurements of airway dimensions and emphysema associated with airflow limitation in heavy smokers: a cross sectional study

Akkelies E. Dijkstra^1^, Dirkje S. Postma^1^, Nick ten Hacken^1^, Judith M. Vonk^1,2^, Matthijs Oudkerk^3^, Peter M.A. van Ooijen^3^, Pieter Zanen^4^, Firdaus A. Mohamed Hoesein^4^, Bram van Ginneken^5^, Michael Schmidt^6^, Harry J.M. Groen^1^

Table S1.

Median AWT_3.5_ and cumulatively assessed perimeter fractions (APF) per lung lobe

| N=492 | AWT_3.5_ (mm) | APF (n) |
| --- | --- | --- |
| Right upper lobe | 0.58 (0.43-0.78) | 40.0 (25.6-59.0) |
| Right middle lobe | 0.55 (0.44-0.72) | 19.9 (11.5-33.1) |
| Right lower lobe | 0.56 (0.43-0.78) | 59.0 (33.6-88.8) |
| Left upper lobe | 0.58 (0.44-0.74) | 43.7 (28.8-61.3) |
| Left lower lobe | 0.61 (0.45-0.81) | 43.5 (23.2-69.0) |
| All lobes | 0.57 (0.44-0.74) | 214.8 (142.4-295.4) |

Data presented as median (interquartile range) values.

*Definition of abbreviations:* AWT_3.5_ = airway wall thickness at 3.5 mm internal lumen size; APF = cumulatively assessed perimeter fractions of airway walls.

The thickest airway walls were present in the left lower lung lobe, i.e. median 0.61 (0.45 - 0.81) mm, and were significantly thicker compared to the airway walls in the other lung lobes (all p-values <0.001). The thinnest airway walls were present in the middle lobe, i.e. median 0.55 (0.44 - 0.72) mm. The highest APF at 3.5 mm internal lumen diameter was observed in the right lower lung lobe, i.e. 59 (34 - 89) and the lowest in the right middle lobe, i.e. 20 (12 - 33).

Table S2. Multivariate linear regression: dependent variable is (A) log transformed AWT_3.5_ and (B) FEV_1_% predicted. LAA%-950 HU was used to quantify the extent of emphysema in this analysis.

| Dependent variable | A. Log-AWT_3.5_ | | B. FEV_1_, % predicted | |
| --- | --- | --- | --- | --- |
|  | Beta | p-value | Beta | p-value |
| FEV_1_,% predicted | -0.010 | <0.001 |  |  |
| Log-AWT_3.5_ |  |  | -30.241 | <0.001 |
| Emphysema; log-%LAA -950 HU | -0.078 | <0.001 | -7.429 | <0.001 |
| Lung volume | -0.048 | <0.001 | 0.006 | 0.993 |
| Pack-years | 0.002 | 0.023 | -0.020 | 0.636 |
| Smoking (former/current) | 0.001 | 0.986 | -2.650 | 0.096 |
| Cough | 0.041 | 0.253 | 0.532 | 0.784 |
| CMH | -0.026 | 0.446 | -2.055 | 0.271 |
| Dyspnea | -0.007 | 0.839 | -2.538 | 0.175 |
| Wheezing | 0.061 | 0.107 | -4.145 | 0.993 |

*Definition of abbreviations:* Log-AWT_3.5_ = log transformed airway wall thickness at 3.5 mm diameter; CMH = chronic mucus hypersecretion; Log-LAA% -950 HU = log transformed percentage of low attenuation areas < -950 HU
